# Society Meetings

**Published:** 1907-10

**Authors:** 


### SOCIETY MEETINGS.

---

THE Southern Surgical and Gynecological Association will hold its next annual meeting at New Orleans, December 17, 18, and 19, 1907, under the presidency of Dr. Howard A. Kelly, of Baltimore. The secretary is Dr. William D. Haggard, of Nashville, who should be addressed concerning places on the program.

---

THE American Hospital Association held its ninth annual conference at Chicago, September 17-20, 1907, under the presidency of Dr. Renwick R. Ross, superintendent of Buffalo General Hospital. A large number of interesting and valuable papers were read and discussed and the meeting was in every sense a succesful one, reflecting great credit upon the distinguished president.

---

THE Medical Association of Central New York will hold its fortieth annual meeting at Rochester, Tuesday, October 15, 1907, under the presidency of Dr. W. B. Jones, of Rochester. Dr. Carlton C. Frederick, of Buffalo, is the first vice-president; Dr. Pascal M. Dowd, of Oswego, is the second vice-president; Dr. C. A. Greenleaf, of Canoga, is the secretary, and Dr. William M. Brown, of Rochester, is the treasurer.

---

THE Sixth District Branch of the Medical Society of the State of New York held its first annual meeting at Ithaca, September 24, 1907, under the presidency of Dr. Ross G. Loop, of Elmira. Dr. C. L. Stiles, of Oswego, was vice-president; Dr. Herbert W. Fudge, of Elmira, was secretary, and Dr. S. A. Mereness, of Milford, was treasurer.

---

THE New York State Conference of Health Officers will hold its next annual meeting at Buffalo, October 14-19, 1907. Every health officer in the state is a member by law and must either attend in person or by substitute. It is estimated that at least 1,200 sanitary officers will be in attendance. It is announced that Governor Hughes will attend the opening meeting. All questions relating to public health will be discussed and the sessions will be open to the public. There will be addresses in convention hall by experts from Washington, Chicago and New York upon the subjects of tuberculosis, pure milk, water filtration, sewage disposal, child-labor, tenement-house problems and many other vital questions. A special feature of the conference will be the tuberculosis and hygienic exhibit at convention hall, which will constitute a museum of preventive medicine showing what is being done in this state for the prevention of disease. The meeting will be held under the auspices of the state department of health of which Dr. Eugene H. Porter is commissioner.

